# A Novel Radiogenomics Biomarker Based on Hypoxic-Gene Subset: Accurate Survival and Prognostic Prediction of Renal Clear Cell Carcinoma

**DOI:** 10.3389/fonc.2021.739815

**Published:** 2021-10-07

**Authors:** Jiahao Gao, Fangdie Ye, Fang Han, Xiaoshuang Wang, Haowen Jiang, Jiawen Zhang

**Affiliations:** ^1^ Department of Radiology, Huashan Hospital, Fudan University, Shanghai, China; ^2^ Department of Urology, Huashan Hospital, Fudan University, Shanghai, China; ^3^ Fudan Institute of Urology, Huashan Hospital, Fudan University, Shanghai, China; ^4^ National Clinical Research Center for Aging and Medicine, Huashan Hospital, Fudan University, Shanghai, China

**Keywords:** clear cell renal cell carcinoma, radiogenomics, contrast-enhanced computed tomography, texture analysis, hypoxia

## Abstract

**Purpose:**

To construct a novel radiogenomics biomarker based on hypoxic-gene subset for the accurate prognostic prediction of clear cell renal cell carcinoma (ccRCC).

**Materials and Methods:**

Initially, we screened for the desired hypoxic-gene subset by analysis using the GSEA database. Through univariate and multivariate cox regression hazard ratio analysis, survival-related hypoxia genes were identified, and a genomics signature was constructed in the TCGA database. Building on this, a hypoxia-gene related radiogenomics biomarker (prediction of hypoxia-genes signature by contrast-enhanced CT radiomics) was constructed in the TCIA-KIRC database by extracting features in the venous phase of contrast-enhanced CT images, selecting features using the mRMR and LASSO algorithms, and building logistic regression models. Finally, we validated the prognostic capability of the new biomarker for patients with ccRCC in an independent validation cohort at Huashan Hospital of Fudan University, Shanghai, China.

**Results:**

The hypoxia-related genomics signature consisting of five genes (IFT57, PABPN1, RNF10, RNF19B and UBE2T) was shown to be significantly associated with survival for patients with ccRCC in the TCGA database, delineated by grouping of the signature expression as either low- or high-risk. In the TCIA database, we constructed a radiogenomics biomarker consisting of 13 radiomics features that were optimal predictors of hypoxia-gene signature expression levels (low- or high-risk) in patients at each institution, that demonstrated AUC values of 0.91 and 0.91 in the training and validation groups, respectively. In the independent validation cohort at Huashan Hospital, our radiogenomics biomarker was significantly associated with prognosis in patients with ccRCC (p=0.0059).

**Conclusions:**

The novel prognostic radiogenomics biomarker that was constructed achieved excellent correlation with prognosis in both the cohort of TCGA/TCIA-KIRC database and the independent validation cohort of Huashan hospital patients with ccRCC. It is anticipated that this work may assist in clinical preferential treatment decisions and promote the process of precision theranostics in the future.

## Introduction

Recognized as the most common urinary cancer, renal cell carcinoma was responsible for more than 175,000 deaths worldwide in 2020 (1, 2). Clear cell renal cell carcinoma (ccRCC) is the predominant pathological type of kidney cancer, accounting for approximately 75% of these malignancies (3). Surgical resection remains the principal treatment for localized or locally advanced renal cell carcinoma. Although surgically resected localized renal cancers have a 5-year survival ranging from 80% - 95%, non-metastatic renal cancers deemed to be high risk have a probability of recurrence or metastasis as high as 30% - 40%, associated with an extremely high mortality rate (4). In recent years, due to widespread use of abdominal ultrasound and CT scans for vague symptoms, an increasing number of serendipitously discovered, asymptomatic renal cancers have been diagnosed. As noted previously, with rather wide variability in the prognosis of individual patients with ccRCC, there is an urgent need for more precise and readily defined prognostic parameters to group patients according to disease risk, to facilitate individualized clinical oncology treatment options.

Tumors are essentially a phenomenon of mutated gene expression as oncogenes and tumor suppressor genes. Genetic heterogeneity of cancer cells is the underlying cause for differences in drug resistance, disease progression, and patient survival (5–7). In contrast to normal cells, the metabolism of glucose by clear cell renal carcinoma cells, even in the presence of sufficient oxygen, is mainly *via* the glycolytic pathway. This ‘pseudo-hypoxia’ was found to be associated with the presence of genetic mutations and deletions in the majority of ccRCC. Hypoxia-inducible factor (HIF)-induced gene mutations promote tumor angiogenesis, metabolic reprogramming, and cancer cell proliferation (8–10). Most HIF targeted-genes are involved in hypoxic responses, and the expression status of hypoxia-related genes exerts a significant impact on the prognosis of patients with ccRCC (11, 12). Although a number of genetic mutations have been found in ccRCC (PBRM1, BAP1, SETD2, and KDM5C), effective and definitive genetic prognostic markers for gene expression in kidney cancer patients are lacking. Additionally, small specimens obtained by puncture biopsy do not accurately reflect the heterogeneity of gene expression across the entire tumor. Reliable information on tumor gene expression is usually obtained by genetic sequencing of surgically resected specimens. Highly invasive and costly for patients, they are not currently available on a large scale and lack universal prognostic guidance.

Radiogenomics is a promising technology in cancer-related research. Based on the use of automated, high-throughput feature extraction methods, it can provide insight into the occurrence, development and heterogeneity of tumors by deeply mining the biological nature of medical images and integrating them with genomic data (13). Likewise, this technique is potentially exceptionally promising for linking highly reproducible, non-invasive imaging features with the disease gene expression profile that is distinctly associated with a clinically meaningful prognosis (14). In recent years, radiogenomics has been reported covering a wide range of tumors, encompassing gene sequences, gene expression, molecular subtypes, and tumor heterogeneity (15–17), effectively providing direction for the formulation of clinical treatment plans, particularly devised for the individual patient.

The current study focused on an important prognostic factor in ccRCC: the association between hypoxia-related gene expression and the prognosis of patients with clear cell renal cell carcinoma. The intent was to develop a prognostic radiogenomics biomarker for ccRCC employing the TCIA-KIRC database, then utilizing radiomics to reflect the expression levels of hypoxia-associated gene subsets through contrast-enhanced CT features, and ultimately validating radiogenomics biomarker on a cohort of ccRCC patients from the Huashan Hospital dataset.

## Materials and Methods

### Datasets

**Figure 1:**
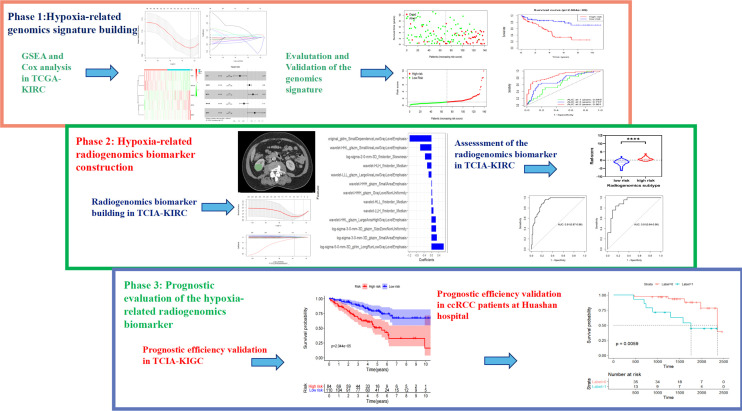
Workflow of the research.

The workflow of the study is depicted in **Figure 1**. The study included three cohorts. Cohort 1 contained transcriptome profiles and corresponding clinical data from TCGA-KIRC with the exception of TCIA, and was divided into training and validation groups. It was used to construct a prognostic risk model and determine the prognostic factors that contributed to hypoxia-related gene mutation. This cohort initially included 318 samples; 41 samples were excluded as there were either no clinical reports or insufficient survival data, leaving 277 samples for analysis. For Cohort 2, clinical and pathological information (including gender, age, T stage, M stage, Stage, Grade) as well as imaging and genetic data were obtained from The Cancer Genome Atlas (TCGA) study website. Multi-institutional medical imaging data were retrospectively obtained from The Cancer Imaging Archive (TCIA) renal clear cell carcinoma database. The data were collected with the permission of each institution’s ethics review committee and de-identified in accordance with the Health Insurance Portability and Accountability Act (HIPAA). Cohort 3 comprised subjects from Huashan Hospital, Fudan University, and the institutional review board approved the study and waived informed consent. Patients diagnosed with ccRCC who had undergone surgical resection with confirmatory pathology between January 2013 and December 2017 were considered for this retrospective study. The optimized risk prediction model based on genomic data was investigated in Cohort 1, and Cohort 2 was used to explore the relationship between hypoxic-related intratumor heterogeneity and the imaging signature. Cohort 3 was used to assess and validate the performance of the radiogenomics prognostic model.

### Imaging Protocol

Cohort 2 initially included 245 patients for whom preoperative baseline abdominal CT or MRI examinations for ccRCC were available. Variations in imaging data in terms of examination modality, machine manufacturer and image acquisition protocol were taken into account. The data were screened according to both inclusion and exclusion criteria. Inclusion criteria included the following: (1) definite postoperative pathology of clear cell renal cell carcinoma with TNM stage; (2) complete imaging data on preoperative enhanced CT or MR scans, with image layer thickness <5 mm, tube voltage 120 KV, and matrix 512*512; and (3) clearly identifiable malignant lesions in the renal parenchymal phase on enhanced CT images. Exclusion criteria included: (1) poor quality enhanced CT images or significant artifacts affecting the region of interest; or (2) inability to successfully extract radiomics features from enhanced CT images. The detailed inclusion procedure is depicted in **Figure S1**.

In Cohort 3, CT examinations of all patients were performed using a 256-row CT system (Brilliance iCT, Philips Medical Systems, The Netherlands). All renal CT images were acquired using a standard three-phase scanning protocol, with parameters as follows: 120 kV; 150-200 mA; rotation time, 0.5-0.75 s; collimation, 128 × 0.625 mm; matrix, 512 × 512; and slice thickness, 1.5 mm. Patients were scanned in the supine position with breath held, inclusive from the top of the diaphragm to the lower edge of the kidney. The abdomen was first scanned, and the enhanced scan utilized a high-pressure syringe injection of a non-ionic contrast agent (1.5 ml/Kg, 3.0 ml/s), with scans at 30 s, 90 s and 300 s following injection to obtain cortical, parenchymal and excretory phase images, respectively. The inclusion and exclusion criteria for Cohort 3 were the same as for Cohort 2.

Cohort 2 initially included 136 samples as a training group from which exclusions included 11 with no obvious tumor, 8 due to poor imaging quality, and 25 with incomplete enhanced CT data. There were 127 males and 67 females with a mean age of 60.45 ± 11.64 years (range, 34 to 88 years). Cohort 3 served as a validation group, derived from Huashan Hospital as an independent prognostic testing dataset. Of 58 initial samples, 8 were excluded due to incomplete enhanced CT imaging data, leaving 50 samples as the final cohort (29 males and 21 females; mean age, 62.9 ± 12.17; range, 33 and 87 years).

### Construction of the Hypoxia-Related Genomics Prognostic Model

The transcriptome data and the corresponding clinical survival data (including age, gender, grade, TMN) of the patients with ccRCC were obtained from the TCGA (https://portal.gdc.cancer.gov/). The hypoxic-associated genes were then obtained from GSEA. Univariate Cox proportional hazard regression analysis collected the candidate hypoxia-related genes, which significantly associated with the overall survival by using R-package “survival” (P < 0.05). Prognostic hypoxic-related genes were divided into risk related genes (hazard ratio, HR >1) and protective related genes (HR <1). The risk score for each patient was then calculated using a linear combination of characteristic gene expression, with characteristic gene expression weighted by their regression coefficients = (expression 1× coefficient gene 1) +(expression 2× coefficient gene 2) +… +(expressing 1× coefficient gene). Patients with ccRCC were divided into high-risk and low-risk groups according to the median risk score.

### Image Processing and Region of Interest Sketching

In Cohorts 2 and 3, the enhanced CT image data were normalized and resized using the z-score method and the mean normalization method. Parenchymal phase CT images of each patient were used for radiomics feature extraction. For each image sequence, a radiologist (14 years of abdominal imaging experience) segmented the lesion contours on each slice using an open-source software (3D Slicer version 4.11.0; Boston, MA). Radiomic features were extracted for each stage of the 3D volume with a python-based radiomics software (Pyradiomics version 3.0.0; https://github.com/Radiomics/pyradiomics) (18). The extracted features are included in **Supplementary Materials I**.

Intra-class and inter-class correlation coefficients (ICC) were used to assess the reliability of the extracted features. A random selection of 50 patients underwent repeat region of interest (ROI) segmentation performed by the same and an additional radiologist (7 years of abdominal imaging experience) 30 days after the initial segmentation. Relevant clinical and pathological information was blinded to the radiologists.

### Radiogenomics Signature Building

In Cohort 2, the extracted radiogenomics features were identified according to the following consecutive steps to construct the radiogenomics model. Initially, features with both intra- and inter- ICC greater than 0.75 were filtered out. Feature dimensionality reduction was further accomplished using the mRMR (minimum Redundancy, Maximum Relevance) method and the Least Absolute Shrinkage and Selection Operator (LASSO) algorithm to select the most optimal and robust features. These features were then combined with their coefficients in the LASSO regression to construct radiogenomics feature labels: Radscores. The Mann-Whitney U test was used to assess the ability of the newly created radiogenomics marker to group patients with different hypoxia gene set expression levels into low- or high-risk groups. We also used ROC curves and area under the curve (AUC) values to evaluate its performance.

We also combined the constructed radiogenomics biomarker with clinical and pathological factors to construct a nomogram which better visualizes the model and increases the reliability and predictive power. First, univariate cox regression analysis was performed for each clinical, pathological variable and radiogenomics biomarker, and then variables with *P*<0.05 in the univariate analysis were included in the multivariate Cox proportional risk model to determine the independent predictors of overall survival (OS). In the multivariate Cox regression, a combined model which consisting radiogenomics biomarker and other useful clinical and pathological factors was built by backward stepwise selection according to the Akaike Information Criterion (AIC), and a nomogram was constructed using R software. Internal validation of the predictive performance of the nomogram was carried out by 1000 resampling with the boots-trap method. ROC curves (1-year, 3-year and 5-year survival) were applied for evaluation. In addition, calibration curves were plotted to ensure the goodness of fit and reliability of the nomogram.

### Radiogenomics Signature Validation

For further validation of the prognostic predictive power of our radiogenomics signature for ccRCC, we used Cohort 3, using the same steps as described previously for image acquisition and radiogenomics features extraction to produce the final radiogenomics scores. Kaplan-Meier curves were produced for overall survival of the patients based on this label, and the log-rank test was used to determine whether the new marker was successful in stratifying the prognosis of the patients. Hazard ratios (HR) and their 95% confidence intervals were obtained to assess survival differences between stratified groups. Univariate Cox regression models were used to further identify whether the radiogenomics signature were independently associated with OS.

### Statistical Analysis

Cohort 1 comprised a training cohort (n=139) and a validation cohort (n=138) for which risk scores were calculated. Using univariate Cox proportional hazard regression analysis, based on the median risk score, the patients in the training cohort were divided into low- and high-risk groups, and the differences between the groups were evaluated and verified. The same approach was performed in the validation cohort, again separating patients into low- and high-risk groups. The radiogenomic features were then collected from the enhanced CT images to determine the relationship between the imaging report and the genome subcloning, intended to predict the patient survival. Finally, we evaluated the prognostic performance of the radiogenomics signatures on the Cohort 3 dataset with enhanced-CT and matched survival data.

The risk model based on hypoxia-related genes proved to be an independent prognostic factor by univariate and multivariate Cox proportional hazard regression analysis. Kaplan-Meier survival curves were constructed to evaluate differences of overall survival between groups. ROC (receiver operating characteristics) curve analysis was performed to evaluate the prediction performance of the risk model and the combined nomogram. Calibration curves were plotted to assess the accuracy and reliability of the combined nomogram. Two-tailed p-values of less than 0.05 were considered statistically significant. Statistical analyses were all performed on R software (version 3.6.3).

## Results

### Phase1: Training of Hypoxia-Related Genomic Subclone Model in Cohort 1

**Figure 2:**
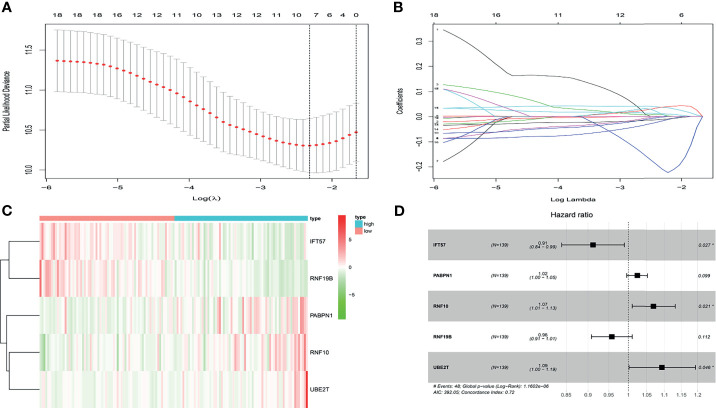
**(A, B)** After univariate logistic regression, the LASSO regression algorithm was performed to further identify the most valuable prognostic hypoxia-related genes with non-zero regression coefficient. **(C)** The heatmap of hypoxia-related prognostic genes expression level. **(D)** The high-risk hypoxia-related prognostic factors selected by multivariate Cox analysis.

**Figure 3:**
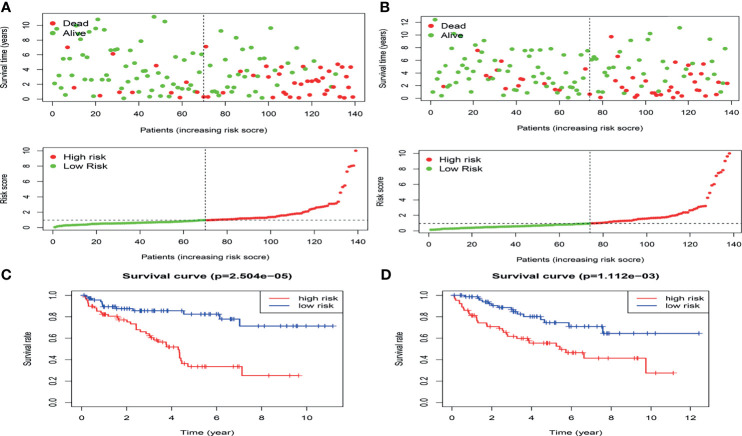
The distribution of the risk scores as well as the relationship between the risk scores and survival status in the training cohort **(A)** and validation cohort**(B)**.The result of K-M analysis for the hypoxia-related risk model in the training cohort **(C)** and validation cohort **(D)**.

In the present study, the risk model was constructed to evaluate hypoxia-related gene status of patients with ccRCC. Initially, a univariate Cox analysis screened prognostic-related hypoxia genes. Then the LASSO Cox regression algorithm was performed to identify the most valuable prognostic hypoxia-related genes with non-zero regression coefficients (**Figures 2A, B**). This produced 5 gene signatures and the risk score of each patient was calculated by performing multivariate Cox analysis. IFT57 and RNF19B were identified as low-risk prognostic genes whereas PABPN1, RNF10 and UBE2T were considered high-risk prognostic factors (**Figures 2C, D**). The detailed information regarding the selected genes are shown in **Table 1**. The Risk score = (0.0234×expression of RABPN1) + (0.0670×expression of RNF10) + (0.0890×expression of UBE2T) – (0.0924×expression of IFT57) – (0.0432×expression of RNF19B). The risk model could be clearly separated from the PCA (Principal Component Analysis) analysis and the genomic signature established was independently correlated with the survival of ccRCC patients (**Supplementary Materials II**). The distribution of the risk scores as well as the relationship between the risk scores and survival data are illustrated in scatterplots (**Figures 3A, B**). Each of the patients with ccRCC was allocated into either a low- or high-risk group according to the median risk score. To comprehensively evaluate the prognostic value of these five gene signatures in the training group, Kaplan-Meier survival curves demonstrated that the patients in the high-risk group had a significantly shorter overall survival compared to patients in the low-risk group. These results were verified by the K-M survival curves in the validation set (**Figures 3C, D**).

**Table 1:** Identification of prognostic hypoxia-related genes in the multivariate cox regression.

Hypoxia-related genes	Coef	HR	HR.95L	HR.95H	Cox p-value
IFT57	-0.092391582	0.911748056	0.840186788	0.989404414	0.026733824
PABPN1	0.023407623	1.023683732	0.995610475	1.052548572	0.098966027
RNF10	0.066942512	1.069234008	1.010052418	1.131883201	0.021208353
RNF19B	-0.043239752	0.957681757	0.907947788	1.010139965	0.112021756
UBE2T	0.089040247	1.093124651	1.001637183	1.192968395	0.04586362

**Figure 4:**
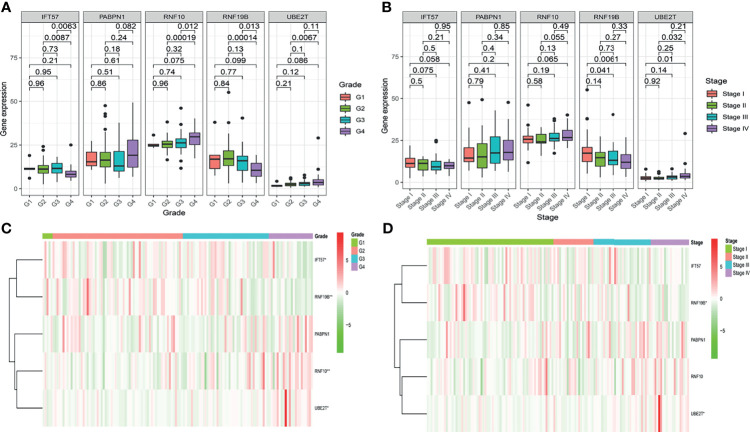
The correlation between the hypoxia-related prognostic genes and clinical pathological, such as Grade **(A, C)** and Stage **(B, D)**, in the training cohort.

To further determine whether the risk-score constituted an independent prognostic factor that correlated with poor prognosis in patients with ccRCC, we performed both univariate and multivariate Cox proportional hazards regression analyses in both the training and validation sets. These revealed that age, stage, and Risk score were significantly associated with the OS of patients with ccRCC in the training set. Critically important, in the validation set, only the Risk score proved to be significantly associated with OS of patients with ccRCC (**Figure S2**). The correlation between the prognostic genes and pathology is shown in **Figure 4**.

### Phase 1: Validation of the Hypoxia-Related Genomics Model in Cohort 2

Based on the hypoxia-related prognostic genes obtained by the above method, we divided Cohort 2 into high- and low-risk groups according to the median value of the Risk score. The low-risk group had a lower death rate and longer survival (**Supplementary Materials IV**). The K-M survival curves indicated that the Risk score was inversely correlated with length of survival (**Figure 7A**). The AUC of the ROC curve of the risk model for overall survival is included in the **Supplementary Materials**.

### Phase 2: Radiomics Feature Selection, Construction of the Radiogenomics Biomarker and Combined Nomogram

**Figure 5:**
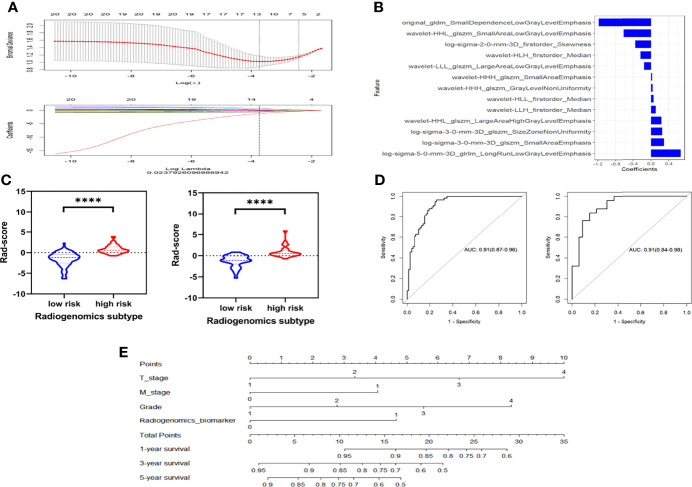
The Lasso regression procedure to select the optimist radiomics features **(A)**. Features selected for hypoxia-related radiogenomics model construction **(B)**. The violin plots of the radiogenomics model in the training cohort and the validation cohort **(C)**. ROC curves shows that the radiogenomics biomarker could exactly distinguish patients into high- an low- hypoxia-related genomics grouping both in the training cohort and validation cohort **(D)**. **(E)** Nomogram of the model incorporating radiogenomics marker and other effective clinical and pathological information through Cox regression. ****p < 0.001.

In Cohort 2, 136 patients were randomized to the training cohort. Enhanced CT images of the renal parenchymal phase from these patients were used to extract radiomics features, starting with 1218 radiomics features. An initial screen of intra- and inter-group ICC > 0.75 reduced the number to 827 features. The mRMR algorithm further downscaled the number of features to 30, following which the most dynamic and relevant features were finally selected for modeling using LASSO regression (**Figure 5A**). The 13 optimal features were combined by multivariate logistic regression for the expression status of the hypoxic gene subgroup, thereby constructing a radiogenomics signature, expressed as radiogenomics score. Details of the included radiomics features are shown in **Figure 5B**. The specific formulae for radiogenomics scores are presented in **Supplementary Materials III**.

We then constructed a combined model incorporating clinical and pathological factors as well as the radiogenomics biomarker. Univariate Cox regression analysis showed that T-stage, M-stage, Grade, Stage and radiogenomics biomarker were associated with survival (*P*<0.05), and these five factors were included in the multivariate Cox regression. After backward stepwise selection, T stage, M stage, Grade and radiogenomics biomarker formed the final combined nomogram (**Figure 5E**). T stage (HR=2.33, *P*=0.02) and radiogenomics biomarker (HR=1.81, *P*=0.03) were shown to be independent predictors of survival (**Table 2**).

**Table 2:** Uni- and multivariable cox regression analysis of predictors of overall survival in cohort 2.

Factors	Univariate Analysis		Multivariate Analysis	
	Hazard Ratio (95%CI)	*P* Value	Hazard Ratio (95%CI)	*P* Value
Age	1.41 (0.84,2.36)	0.19	NA	NA
Gender	1.36 (0.81,2.28)	0.25	NA	NA
T stage	3.97 (2.27,6.94)	<0.01	2.33 (1.21,4.50)	0.02
M stage	3.94 (2.30,6.76)	<0.01	1.67 (0.87,3.24)	0.12
Grade	2.28 (1.57,3.30)	0.03	1.42 (0.93,2.18)	0.10
Stage	3.17 (2.05,4.92)	<0.01	NA	NA
Radiogenomics biomarker	2.46 (1.45,4.19)	<0.01	1.81 (1.04,3.14)	0.03

NA, not available.

### Phase 2: Radiogenomics Model and Combined Nomogram Evaluation

The Mann-Whitney U test vividly demonstrates the ability of the radiogenomics signature to group hypoxic gene subsets, as depicted in a violin plot (**Figure 5C**). Shown in **Figure 5D**, this Radiogenomics score in the ROC curves discriminated well between high- and low-risk groups for the subset of hypoxia genes. The AUC values of the ROC curves reached 0.91 in the training group and 0.91 in the validation group.

The ROC curve demonstrated that the combined nomogram incorporating clinical and pathological factors and the radiogenomics biomarker was effective in predicting 1-year, 3-year and 5-year survival rates (AUC=0.789, 0.782, 0.731 respectively) in cohort 2 (**Supplementary Materials V**). It could further improve the predictive effectiveness of the model compared to the Radiogenomics biomarker alone. In addition, the calibration curves of the nomogram for predicting survival are close to the “actual curves” (**Supplementary Materials V**), indicating that the model fits well and is reliable.

**Figure 6:**
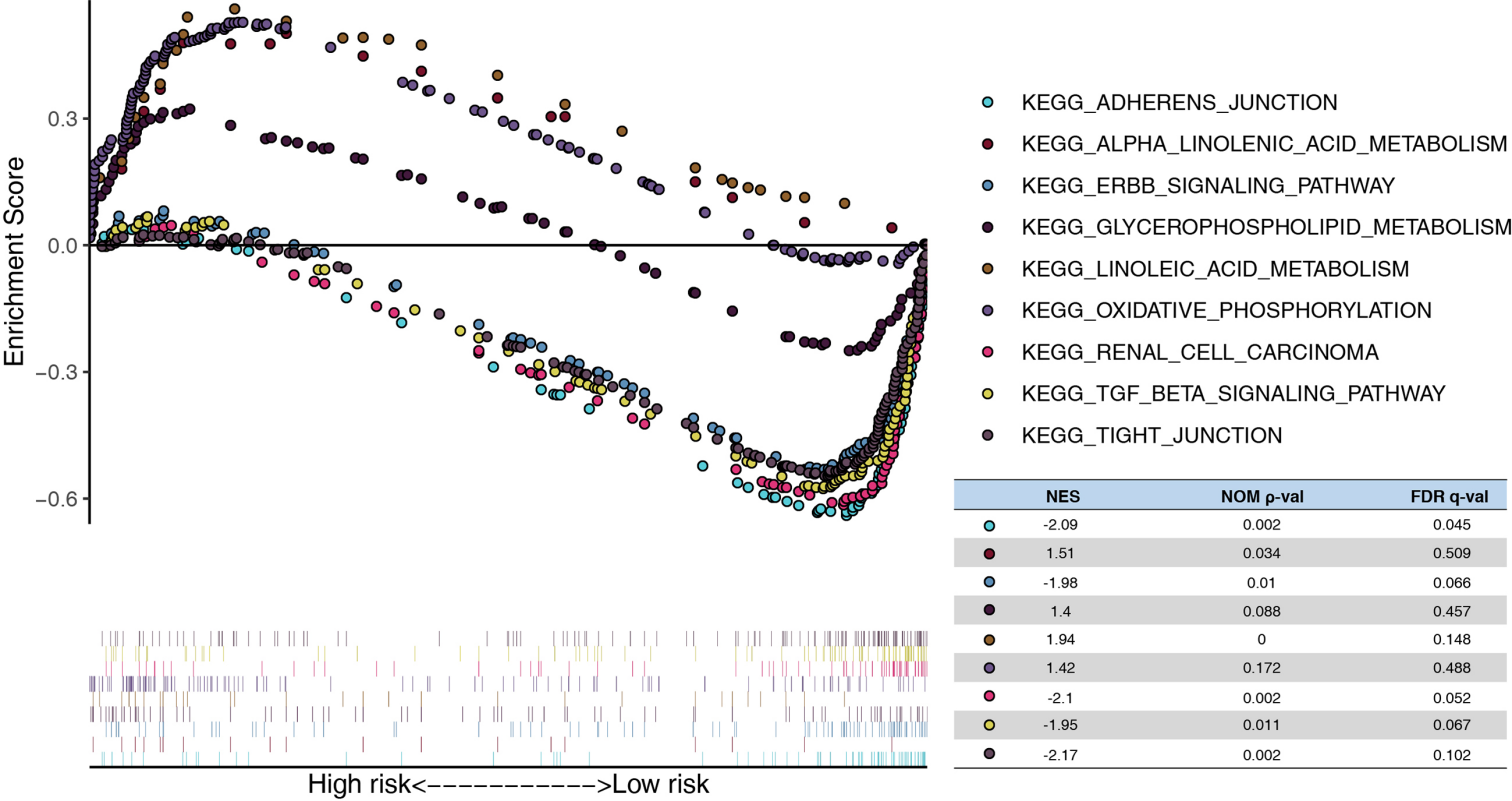
Functional analysis of the risk model. GSEA analysis between high- and low-risk in TCGA. NES, normalized enrichment score; Nom, nominal; FDR, false discovery rate.

In addition, the GSEA demonstrated that linoleic acid/alpha linolenic/glycerophospholipid metabolism and oxidative phosphorylation were significantly enriched in the high-risk model, whereas tight/adherens junction, renal cell carcinoma, ERBB signaling pathway, and the TGF beta signature pathway were enriched in the low-risk model. (**Figure 6**)

### Phase 3: Validation of the Prognostic Predictive Performance for the Radiogenomics Biomarker

For the independent validation group, extraction of radiomics features needed to construct the radiogenomics model, and the radiogenomics score were determined for each patient in like manner to the techniques reviewed previously. Based on the best cut-off values of the radiogenomics scores in Cohort 2, 14 patients were included in the predicted-high risk group of hypoxic gene subset, while 36 patients were included in the low-risk group.

**Figure 7:**
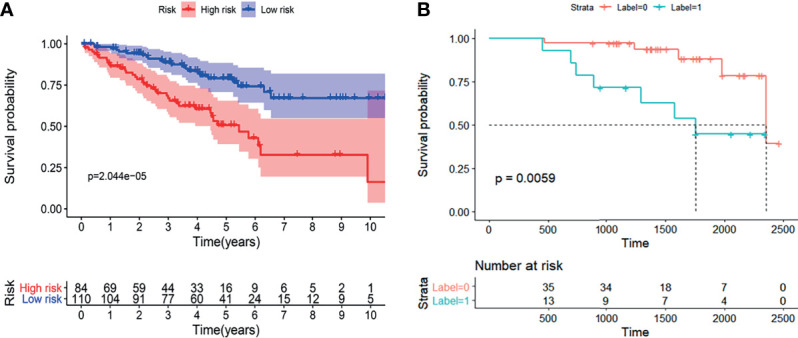
The K-M analysis showed that the established radiogenomics biomarker could successfully divide ccRCC patients in to high- and low-risk, and possessing effective prognostic predictive ability both in the TCIA-KIRC **(A)** database and Huashan validation database **(B)**.

The results demonstrated that the radiogenomics signature was associated with overall survival (P <0.01) (**Figure 7B**). Univariable Cox regression analysis revealed that the radiogenomics biomarker proved to be an independent preoperative prognostic factor in patients with ccRCC (HR=1.57, *P <*0.01).

## Discussion

In this study, we focused on establishing the relationship between hypoxia gene expression and prognosis of patients with ccRCC. Our data confirmed that high- and low-risk grouping of hypoxia genes were significantly associated with prognosis. Furthermore, a non-invasive, efficient assessment of the expression level of hypoxic gene sets was performed utilizing a radiomics approach. Finally, this association of genomics and radiomics was verified in the validation set of ccRCC patients, and survival statistics established that our radiogenomics biomarker was capable of stratifying the prognosis of ccRCC patients. In addition, we combined A with validated clinical and pathological factors to build an integrated model and visualized it by means of a nomogram, further improving the reliability and predictive efficacy of the model. The reliability of the radiogenomics biomarker as an independent prognostic marker for ccRCC was further demonstrated in the multivariate COX regression. It would seem appropriate for this biomarker to serve as a non-invasive prognostic marker for ccRCC that could facilitate individualized, potentially targeted treatment of tumors.

A high frequency of VHL gene mutations has been observed in up to 80% of ccRCC. The function of the VHL protein (pVHL) has been well analyzed (9, 10). pVHL contributes to the ubiquitination and degradation of hypoxia-inducible factor (HIF), a transcription factor of VEGF, under hypoxic conditions. In contrast, mutations in the VHL gene lead to reduced degradation of HIF, and may lead to VEGF overexpression and angiogenesis, which in turn lead to the development of ccRCC. Therefore, the expression levels of hypoxia-associated gene sets are significantly associated with the prognosis of patients with ccRCC (19–21). Intratumoral heterogeneity has been cited as a cause of prognostic differences of individual patients, which is reflected by gene expression data. Methods currently in common use to describe tumor heterogeneity include genetic testing and histopathology. Such approaches confirm the heterogeneity of cancer at the genetic level and can yield greater insight into the establishment of effective treatments. However, they are often expensive and invasive. Moreover, the biological characteristics of the excised tumor tissue may differ somewhat from the tumor as a whole, thereby misrepresenting the actual tumor hypoxic gene expression.

Surgery remains the preferred treatment modality for limited kidney cancer as it lacks sensitivity to conventional radiotherapy and chemotherapy. For inoperable patients with intermediate to advanced ccRCC, targeted therapies and immunotherapy are often recommended. However, the heterogeneity of tumors between patients leads to extremely variable treatment responses, and current clinicopathological markers such as Fuhrman grading and TNM staging do not adequately or effectively reflect these biological differences. Clearly, novel independent prognostic biomarkers are needed to predict prognosis of ccRCC (22, 23). Genetic mutations are the principal initiators of tumor cell heterogeneity, and variations in the tumor gene set will continue to accumulate further altering the genetic profile. Therefore, many studies have been conducted to establish genetic biomarkers to predict the prognosis of ccRCC, and these markers have been associated with various physiological changes in the tumor, such as immune infiltration and metabolic changes, and have achieved good predictive results (24–26). Among these, hypoxia-associated genes have been important factors in the development of KIRC as well as predicting prognosis. An alternative method for this type of research is the use of gene chips, but their cost limits clinical application, and there remains a need for efficient, non-invasive and cost-effective clinical biomarkers.

In recent years, with the deep application of artificial intelligence in the field of image processing, radiomics has developed rapidly. Despite tumor imaging being principally limited to morphological features in routine clinical practice, the ability of imaging to provide a comprehensive view of the spatial-temporal heterogeneity of individual tumors is unmatched by other biomarkers and examination modalities. Radiomics can provide non-invasive tools to study tumor biology, capture tumor heterogeneity, and monitor tumor evolution and response to treatment. There have been numerous radiomics studies applied to the differential diagnosis, molecular typing and prognostic prediction of ccRCC (27–29). Unfortunately, neither the computational features extracted by traditional radiomics using machine-learning methods, nor the “black-box” selection of deep learning methods can explain the function of radiomics for the prediction of clinical events. The interpretability of these methods is not strong, and in particular, explanations of the underlying biological and molecular mechanisms are lacking. In contrast, radiogenomics profoundly reflects the nature of tumor heterogeneity and fully explains the latent causes affecting prognostic survival. Both excellent predictive results are achieved through radiogenomics approaches and the biology of this novel biomarker can be summarized in terms of the essence of cancer pathogenesis: relevant gene mutations, thus effectively improving the interpretability of the model and facilitating its future clinical application.

This study has some limitations: (1) the TCIA database contains imaging data from multiple centers with various imaging machines and image acquisition protocols. Although strict inclusion and exclusion criteria were used and the model was validated in an independent center, the results need to be further investigated in future clinical trials in more centers. (2) The region of interest was extracted in the venous phase and segmented manually during the radiomics feature extraction process, which may lack the heterogeneity of tumor features embedded in other phases of CT scans and may have some selection bias. (3) Different combinations of machine learning algorithms other than multivariate logistic regression and COX regression need to be considered in the future to integrate and optimize the best models for more effective feature selection and to improve model prediction performance. (4) More clinical and pathological factors may need to be incorporated in the future to improve the predictive power and reliability of the model.

## Conclusions

In this study, a novel radiogenomics biomarker based on the expression of hypoxic gene subset was developed for the accurate prediction of prognosis in ccRCC. This new biomarker achieved good survival prediction in both the training set of TCIA/TCGA and the independent validation set of Huashan hospital and may assist in clinical preferential treatment decisions for ccRCC in the future and promote the process of precision prognosis and treatment.

## Data Availability Statement

The original contributions presented in the study are included in the article/**Supplementary Materials**. Further inquiries can be directed to the corresponding authors.

## Ethics Statement

The studies involving human participants were reviewed and approved by the Institutional Review Board of Huashan Hospital, Fudan University. The patients/participants provided their written informed consent to participate in this study. Written informed consent was obtained from the individual(s) for the publication of any potentially identifiable images or data included in this article.

## Author Contributions

JG, FY, and FH contributed equally to this work. Study design and concept, JG and FY. Provision of study materials or patients, FH. Data analysis and interpretation, FH and XW. Supervision, JZ and HJ. Manuscript writing: all authors. Final approval of manuscript: all authors. All authors contributed to the article and approved the submitted version.

## Funding

This work was funded by the National Natural Science Foundation of China (82071877), National Key Research and Development Project in China (No. 2017YFC0113405), and Launching Fund of Huashan Hospital (North Hospital) Affiliated to Fudan University (No. HSBY2020003).

## Conflict of Interest

The authors declare that the research was conducted in the absence of any commercial or financial relationships that could be construed as a potential conflict of interest.

## Publisher’s Note

All claims expressed in this article are solely those of the authors and do not necessarily represent those of their affiliated organizations, or those of the publisher, the editors and the reviewers. Any product that may be evaluated in this article, or claim that may be made by its manufacturer, is not guaranteed or endorsed by the publisher.

## References

[1] Siegel RL Miller KD Fuchs HE Jemal A. Cancer Statistics, 2021. CA Cancer J Clin (2021) 71:7–33. doi: 10.3322/caac.21654 33433946

[2] Sung H Ferlay J Siegel RL Laversanne M Soerjomataram I Jemal A. Global Cancer Statistics 2020: GLOBOCAN Estimates of Incidence and Mortality Worldwide for 36 Cancers in 185 Countries. CA Cancer J Clin (2021) 71:209–49. doi: 10.3322/caac.21660 33538338

[3] Gupta K Miller JD Li JZ Russell MW Charbonneau C. Epidemiologic and Socioeconomic Burden of Metastatic Renal Cell Carcinoma (mRCC): A Literature Review. Cancer Treat Rev (2008) 34:193–205. doi: 10.1016/j.ctrv.2007.12.001 18313224

[4] Lam JS Shvarts O Leppert JT Pantuck AJ Figlin RA Belldegrun AS. Postoperative Surveillance Protocol for Patients With Localized and Locally Advanced Renal Cell Carcinoma Based on a Validated Prognostic Nomogram and Risk Group Stratification System. J Urol (2005) 174:466–72, 472, 801. doi: 10.1097/01.ju.0000165572.38887.da 16006866

[5] Moore AL Batavia AA Kuipers J Singer J Burcklen E Schraml P. Spatial Distribution of Private Gene Mutations in Clear Cell Renal Cell Carcinoma. Cancers (2021) 13:209–49. doi: 10.3390/cancers13092163 PMC8124666 33946379

[6] Marostica E Barber R Denize T Kohane IS Signoretti S Golden JA. Development of a Histopathology Informatics Pipeline for Classification and Prediction of Clinical Outcomes in Subtypes of Renal Cell Carcinoma. Clin Cancer Res (2021) 27:2868–78. doi: 10.1158/1078-0432.CCR-20-4119 33722896

[7] Ross-Macdonald P Walsh AM Chasalow SD Ammar R Papillon-Cavanagh S Szabo PM. Molecular Correlates of Response to Nivolumab at Baseline and on Treatment in Patients With RCC. J Immunother Cancer (2021) 9:1506–20. doi: 10.1136/jitc-2020-001506 PMC7931766 33658305

[8] Akhtar M Al-Bozom IA Al Hussain T. Molecular and Metabolic Basis of Clear Cell Carcinoma of the Kidney. Adv Anat Pathol (2018) 25:189–96. doi: 10.1097/PAP.0000000000000185 29465421

[9] Na X Wu G Ryan CK Schoen SR di'Santagnese PA Messing EM. Overproduction of Vascular Endothelial Growth Factor Related to Von Hippel-Lindau Tumor Suppressor Gene Mutations and Hypoxia-Inducible Factor-1 Alpha Expression in Renal Cell Carcinomas. J Urol (2003) 170:588–92. doi: 10.1097/01.ju.0000074870.54671.98 12853836

[10] Igarashi H Esumi M Ishida H Okada K. Vascular Endothelial Growth Factor Overexpression is Correlated With Von Hippel-Lindau Tumor Suppressor Gene Inactivation in Patients With Sporadic Renal Cell Carcinoma. Cancer-Am Cancer Soc (2002) 95:47–53. doi: 10.1002/cncr.10635 12115316

[11] Liu Z Sun T Piao C Zhang Z Kong C. METTL13 Inhibits Progression of Clear Cell Renal Cell Carcinoma With Repression on PI3K/AKT/mTOR/HIF-1α Pathway and C-Myc Expression. J Transl Med (2021) 19:209. doi: 10.1186/s12967-021-02879-2 33985542 PMC8120818

[12] Choueiri TK Bauer TM Papadopoulos KP Plimack ER Merchan JR McDermott DF. Inhibition of Hypoxia-Inducible Factor-2α in Renal Cell Carcinoma With Belzutifan: A Phase 1 Trial and Biomarker Analysis. Nat Med (2021) 27:802–5. doi: 10.1038/s41591-021-01324-7 PMC9128828 33888901

[13] Fan M Xia P Clarke R Wang Y Li L. Radiogenomic Signatures Reveal Multiscale Intratumour Heterogeneity Associated With Biological Functions and Survival in Breast Cancer. Nat Commun (2020) 11:4861. doi: 10.1038/s41467-020-18703-2 32978398 PMC7519071

[14] Lin P Lin Y Gao R Wen R Qin H He Y. Radiomic Profiling of Clear Cell Renal Cell Carcinoma Reveals Subtypes With Distinct Prognoses and Molecular Pathways. Transl Oncol (2021) 14:101078. doi: 10.1016/j.tranon.2021.101078 33862522 PMC8065300

[15] Calmon R Dangouloff-Ros V Varlet P Deroulers C Philippe C Debily M. Radiogenomics of Diffuse Intrinsic Pontine Gliomas (DIPGs): Correlation of Histological and Biological Characteristics With Multimodal MRI Features. Eur Radiol (2021) 1–12. doi: 10.1007/s00330-021-07991-x 34003354

[16] Kirienko M Sollini M Corbetta M Voulaz E Gozzi N Interlenghi M. Radiomics and Gene Expression Profile to Characterise the Disease and Predict Outcome in Patients With Lung Cancer. Eur J Nucl Med Mol Imaging (2021) 48:3643–55. doi: 10.21203/rs.3.rs-157951/v1 PMC8440255 33959797

[17] Singh G Manjila S Sakla N True A Wardeh AH Beig N. Radiomics and Radiogenomics in Gliomas: A Contemporary Update. Br J Cancer (2021) 125:641–57. doi: 10.1038/s41416-021-01387-w PMC8405677 33958734

[18] van Griethuysen JJM Fedorov A Parmar C Hosny A Aucoin N Narayan V. Computational Radiomics System to Decode the Radiographic Phenotype. Cancer Res (2017) 77:e104–7. doi: 10.1158/0008-5472.CAN-17-0339 PMC5672828 29092951

[19] Lidgren A Hedberg Y Grankvist K Rasmuson T Bergh A Ljungberg B. Hypoxia-Inducible Factor 1alpha Expression in Renal Cell Carcinoma Analyzed by Tissue Microarray. Eur Urol (2006) 50:1272–7. doi: 10.1016/j.eururo.2006.05.043 16814458

[20] Schindl M Schoppmann SF Samonigg H Hausmaninger H Kwasny W Gnant M. Overexpression of Hypoxia-Inducible Factor 1alpha is Associated With an Unfavorable Prognosis in Lymph Node-Positive Breast Cancer. Clinical Cancer Res (2002) 8:1831–7. 12060624

[21] Birner P Schindl M Obermair A Plank C Breitenecker G Oberhuber G. Overexpression of Hypoxia-Inducible Factor 1alpha is a Marker for an Unfavorable Prognosis in Early-Stage Invasive Cervical Cancer. Cancer Res (2000) 60:4693–6. 10987269

[22] Zhou F Shen D Xiong Y Cheng S Xu H Wang G. CTHRC1 Is a Prognostic Biomarker and Correlated With Immune Infiltrates in Kidney Renal Papillary Cell Carcinoma and Kidney Renal Clear Cell Carcinoma. Front Oncol (2020) 10:570819. doi: 10.3389/fonc.2020.570819 33628726 PMC7898899

[23] Gkagkalidis K Kampantais S Dimitriadis G Gourvas V Kapoukranidou D Mironidou-Tzouveleki M. Expression of HIF-2a in Clear-Cell Renal Cell Carcinoma Independently Predicts Overall Survival. Med Mol Morphol (2020) 53:229–37. doi: 10.1007/s00795-020-00249-3 32219604

[24] Fan X Liu B Wang Z He D. TACC3 is a Prognostic Biomarker for Kidney Renal Clear Cell Carcinoma and Correlates With Immune Cell Infiltration and T Cell Exhaustion. Aging (2021) 13:8541–62. doi: 10.18632/aging.202668 PMC8034911 33714201

[25] Yang F Zhao G Ge L Song Y Hong K Zhang H. Identification of a Two-M6a RNA Methylation Regulator Risk Signature as an Independent Prognostic Biomarker in Papillary Renal Cell Carcinoma by Bioinformatic Analysis. BioMed Res Int (2021) 2021:4582082. doi: 10.1155/2021/4582082 33628782 PMC7884118

[26] Niu X Zhu Z Shao E Bao J. ACE2 Is a Prognostic Biomarker and Associated With Immune Infiltration in Kidney Renal Clear Cell Carcinoma: Implication for COVID-19. J Oncol (2021) 2021:8847307. doi: 10.1155/2021/8847307 33564310 PMC7849311

[27] Raman SP Chen Y Schroeder JL Huang P Fishman EK. CT Texture Analysis of Renal Masses: Pilot Study Using Random Forest Classification for Prediction of Pathology. Acad Radiol (2014) 21:1587–96. doi: 10.1016/j.acra.2014.07.023 PMC4352301 25239842

[28] Chandarana H Rosenkrantz AB Mussi TC Kim S Ahmad AA Raj SD. Histogram Analysis of Whole-Lesion Enhancement in Differentiating Clear Cell From Papillary Subtype of Renal Cell Cancer. Radiology (2012) 265:790–8. doi: 10.1148/radiol.12111281 23175544

[29] Juntu J Sijbers J De Backer S Rajan J Van Dyck D. Machine Learning Study of Several Classifiers Trained With Texture Analysis Features to Differentiate Benign From Malignant Soft-Tissue Tumors in T1-MRI Images. J Magn Reson Imaging (2010) 31:680–9. doi: 10.1002/jmri.22095 20187212

