# Supplementary material for: A Novel Radiogenomics Biomarker Based on Hypoxic-Gene Subset: Accurate Survival and Prognostic Prediction of Renal Clear Cell Carcinoma
Source: Front Oncol. 2021 Oct 7;11:739815. doi: 10.3389/fonc.2021.739815 (PMC8529272; doi:10.3389/fonc.2021.739815)
Supplement: Supplementary file 1 [file DataSheet_1.doc]

**Supplementary Material**

**I. Radiomics features extraction methodology**

**II.** **Genomics model construction and performance evaluation in TCGA-KIRC**

**III.** **Calculation formula of the radiogenomics biomarker.**

**IV. Validation of the genomics signature in TCIA-KIRC.**

**V. Construction, performance and validation of the combined nomogram**

**VI. R packages we used in this study**

**Fig. S1.** Recruitment pathway for patients in this study.

**Fig. S2.** Performance of the hypoxia-related genomics signature in TCIA-KIRC

**Fig. S3.** Performance of the identified gene signature and the radiomics signature. (A-B) ROC analysis in raining cohort and validation cohort to validate the performance of the gene signature for 1-3-and 5-years.

**Fig. S4.** The distribution of the risk scores as well as the relationship between the risk scores and survival status in TCIA-KIRC (A) .The result of K-M analysis for the hypoxia-related risk model in TCIA-KIRC (B) .

**Fig. S5.** ROC analysis in TCIA-KIRC to validate the performance of the gene signature for 1-3-and 5-years.

**Fig. S6.** The correlation between the hypoxia-related prognostic genes and clinical pathological, such as Grade (A,C) and Stage (B,D), in the training cohort.

**Fig. S7.** Performance of the combined nomogram. ROC analysis in Cohort 2 to validate the performance of the combined nomogram for 1-,3-and 5-years.

**Fig. S8.** Calibration curves of the combined nomogram for 3-year(A) and 5-year(B) survival in Cohort 2.

**I. R****adiomic****s features extraction methodology**

1218 radiomics features were extracted from the contrast-enhanced CT scans (including plain scan, arterial phase and venous phase). The radiomics features can be divided into six categories:(1) Shape features (n=14); (2) First Order Statistics (n=18); (3) Gray Level Cooccurrence Matrix (GLCM) features (n=22); (4) Gray Level Run Length Matrix (GLRLM) features (n=16); (5) Grey Level Size Zone Matrix (GLSZM) features (n=16); (6) Gray Level Dependence Matrix (GLDM) features (n=14). After 5 times of wavelet filter transform and 8 times of Laplacian of Gaussian (LoG) transform, all kinds of features except shape features increased by 14 times (1+5+8). Therefore, the total number of features extracted for each phase are:14*1(shape features)+(18+22+16+16+14)*14=1218.

The specific contents of each type of features could be seen on the website: <https://pyradiomics.readthedocs.io/en/latest/features.html.> Then we performed the minimum redundancy maximum relevance (mRMR) algorithm to further select the features. 30 characteristics that were most correlated with the results and least correlated with each other were selected for the LASSO regression. Finally, the radiogenomics signatures was constructed by the LASSO logistic regression model. The radiogenomics biomarker were composed of the features with non-zero coefficients in the LASSO regression, through a linear combination of their weighted coefficients.


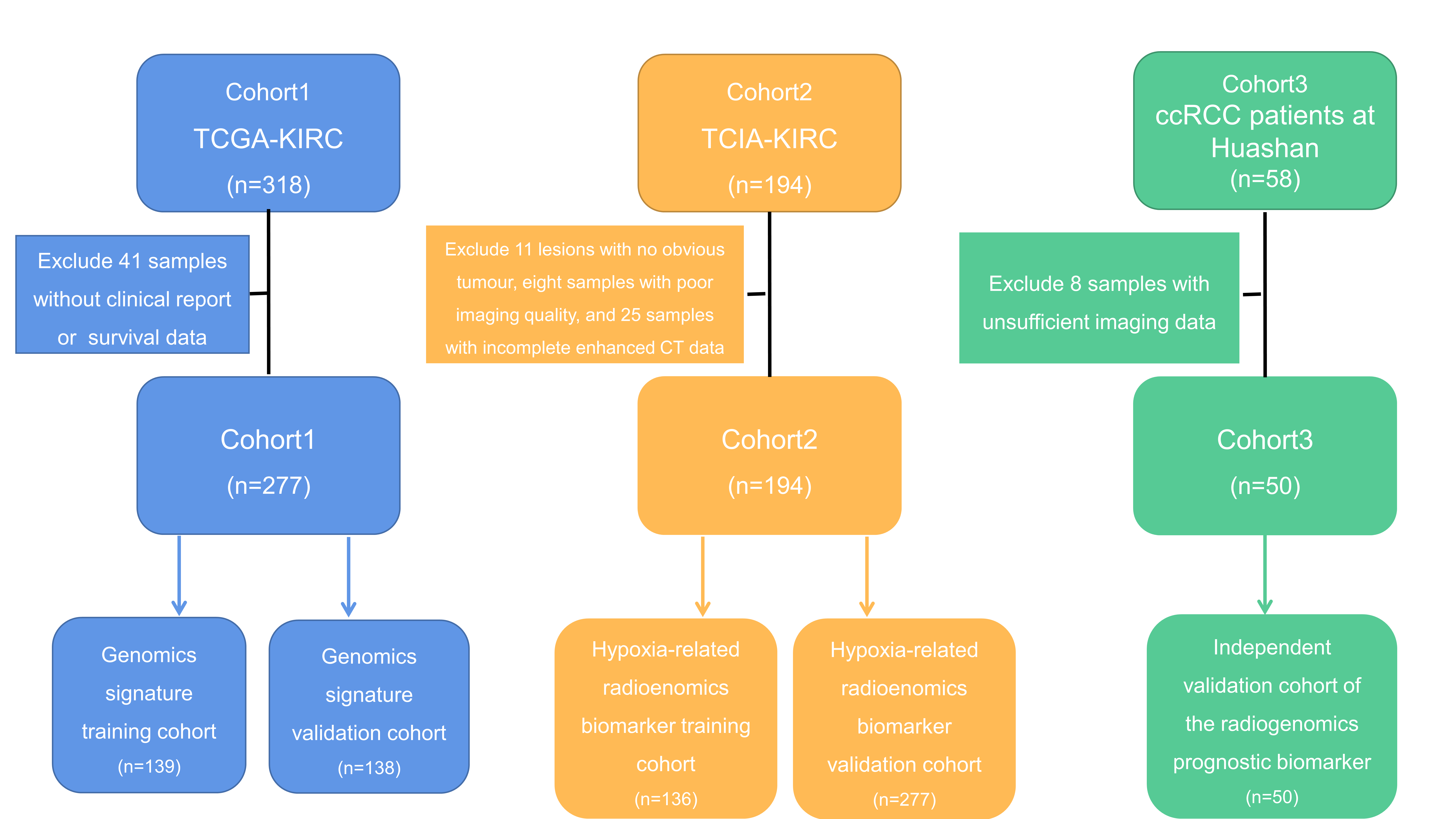


**Fig. S1.** Recruitment pathway for patients in this study

**II.** **Genomics model construction and performance evaluation in TCGA-KIRC**

**(1) Independent validation of the riskScore (hypoxia-related genomics signature) in TCGA-KIRC.**


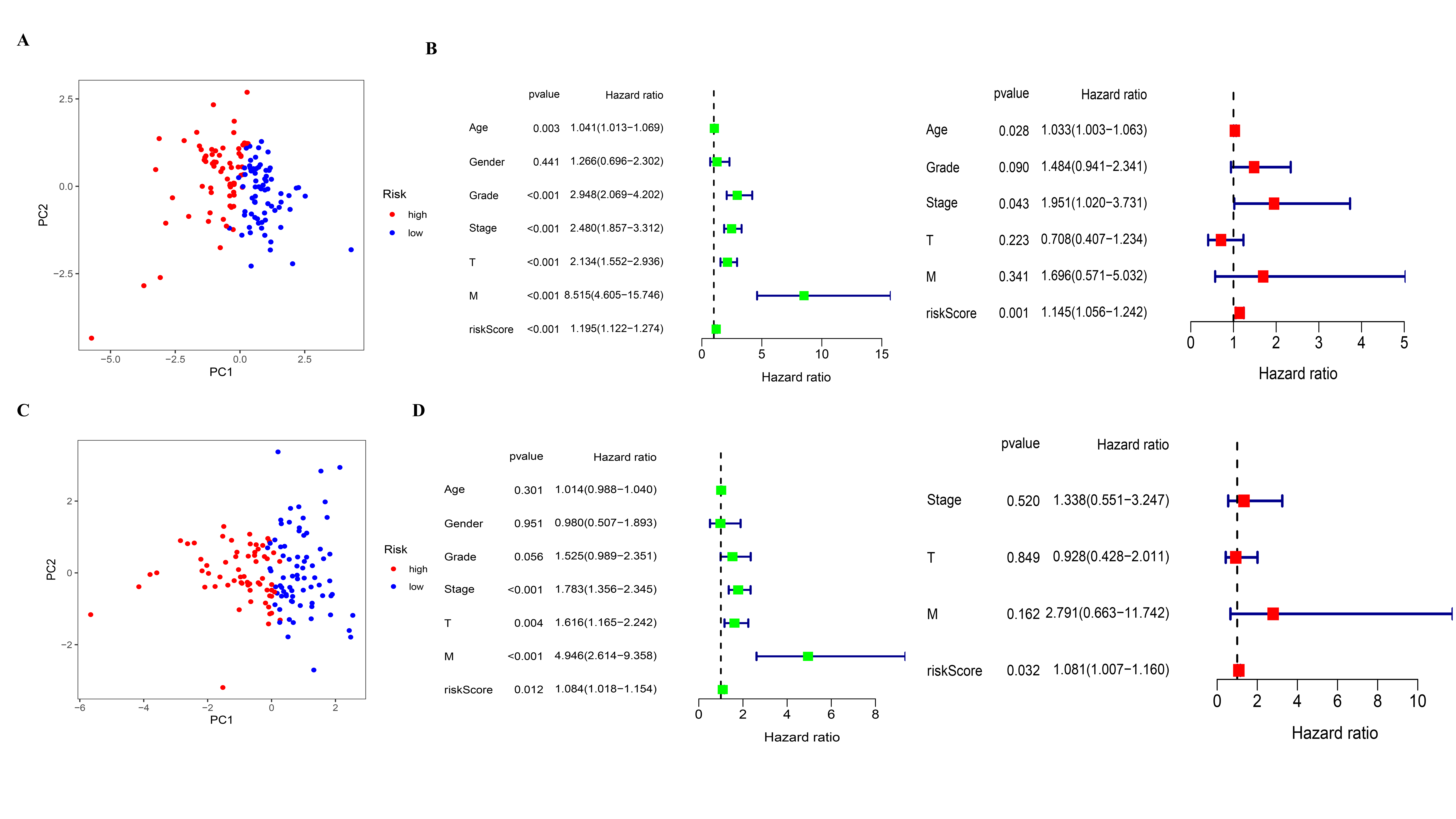


**Fig. S2.** Hypoxia-related genes could distinguish patients into different genomic subclone. (A,D)PCA of the genomic subclone in training cohort and validation cohort. (B-C/E-F) Univariate and multivariate Cox regression analysis of the clinical features and genomic feature for overall survival of ccRCC.PCA:principal component analysis

1. **Prognostic evaluation of the genomics signature in TIGA-KIRC, by ROC curve and AUC value.**

**
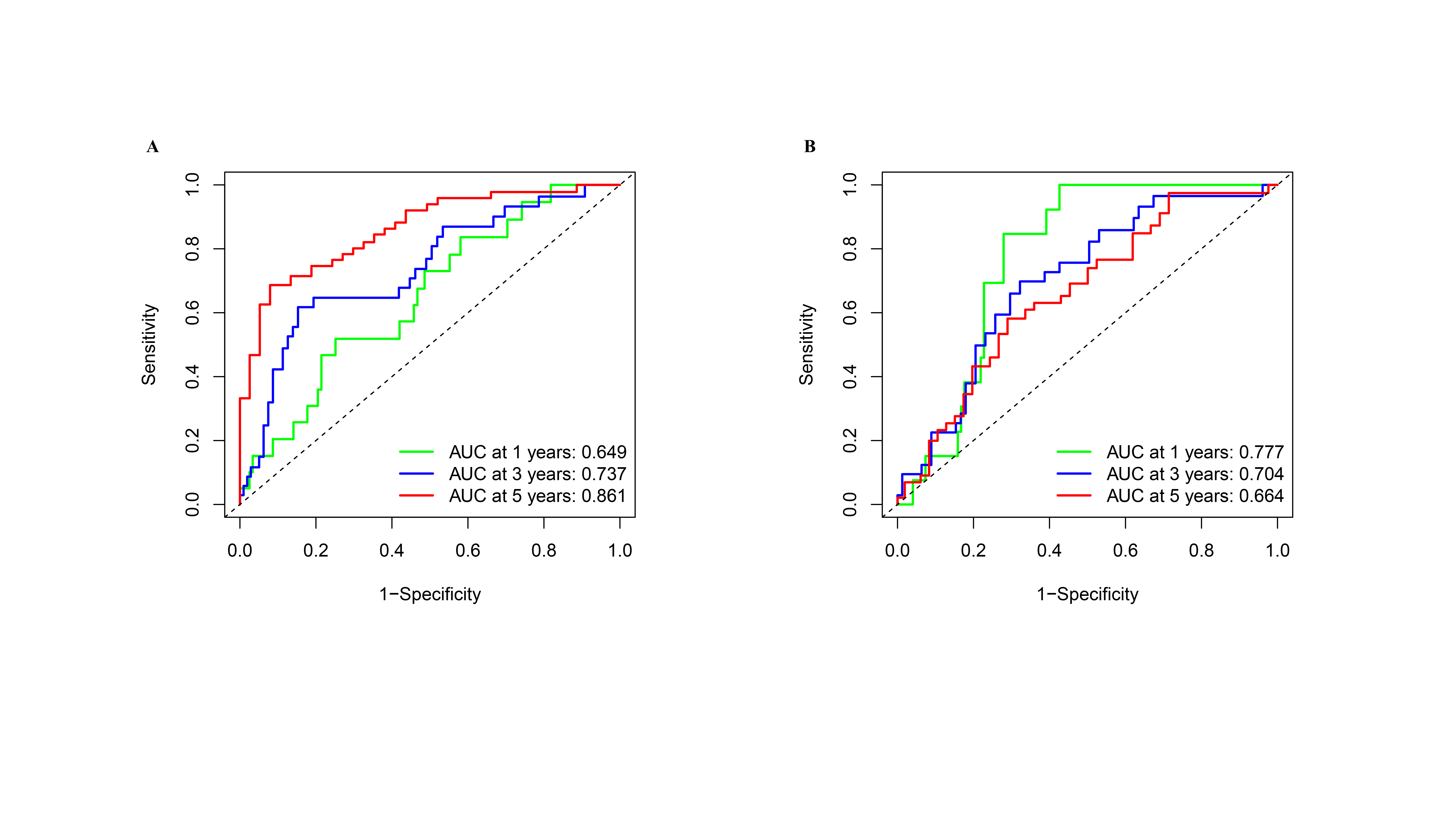
**

**Fig. S3.** Performance of the identified gene signature and the radiomics signature. (A-B) ROC analysis in training cohort and validation cohort to validate the performance of the gene signature for 1-3-and 5-years.

**III. Calculation formula of the radiogenomics biomarker.**

***Radiogenomic-score***== -0.61*wavelet-HHL_glszm_SmallAreaLowGrayLevelEmphasis

-1.183*original_gldm_SmallDependenceLowGrayLevelEmphasis

-0.348*log-sigma-2-0-mm-3D_firstorder_Skewness

+0.661*log-sigma-5-0-mm-3D_glrlm_LongRunLowGrayLevelEmphasis

+0.249*log-sigma-3-0-mm-3D_glszm_SizeZoneNonUniformity

+0.027*wavelet-HHH_glszm_SmallAreaEmphasis

+0.233*wavelet-HHL_glszm_LargeAreaHighGrayLevelEmphasis

+0.291*log-sigma-3-0-mm-3D_glszm_SmallAreaEmphasis

-0.234*wavelet-HLH_firstorder_Median

-0.151*wavelet-LLL_glszm_LargeAreaLowGrayLevelEmphasis

+0.108*wavelet-LLH_firstorder_Median

+0.059*wavelet-HLL_firstorder_Median+0.039*wavelet-HHH_glszm_GrayLevelNonUniformity

-0.531

**IV.Validation of the genomics signature in TCIA-KIRC.**

**
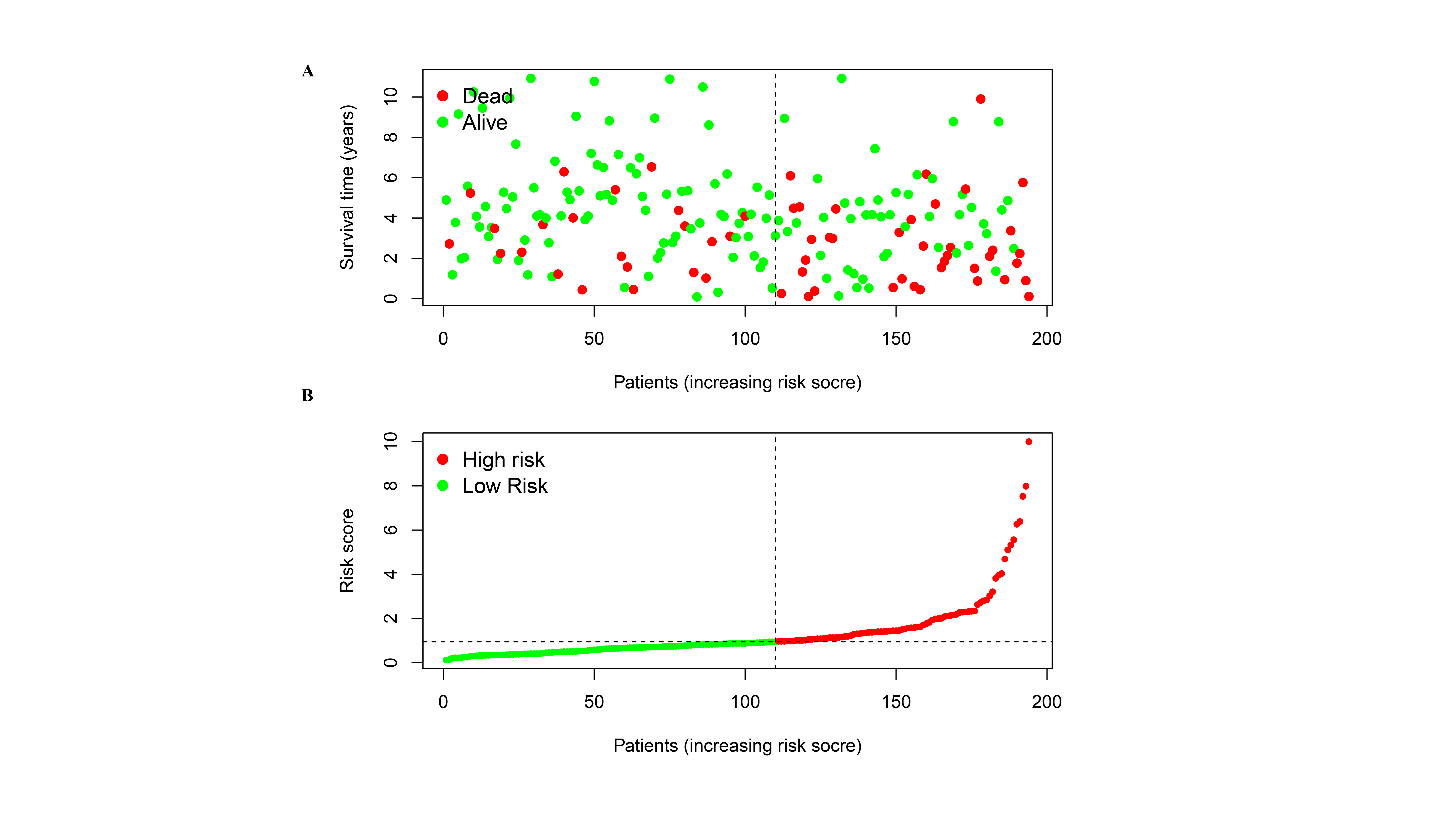
Fig. S4.** The distribution of the risk scores as well as the relationship between the risk scores and survival status in TCIA-KIRC (A, B) .

**
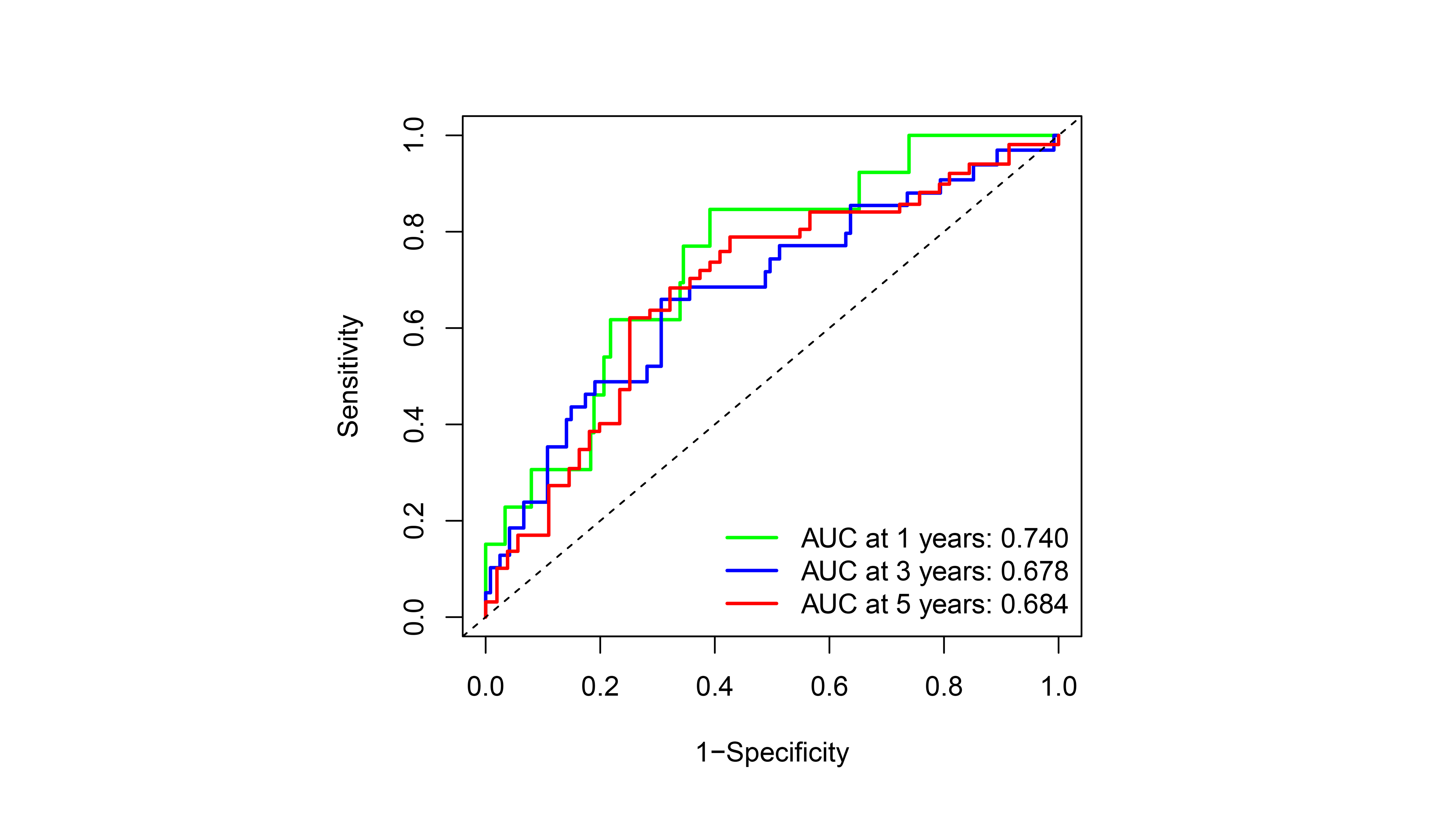
Fig. S5.** ROC analysis in TCIA-KIRC to validate the performance of the gene signature for 1-, 3-,and 5-years.

**
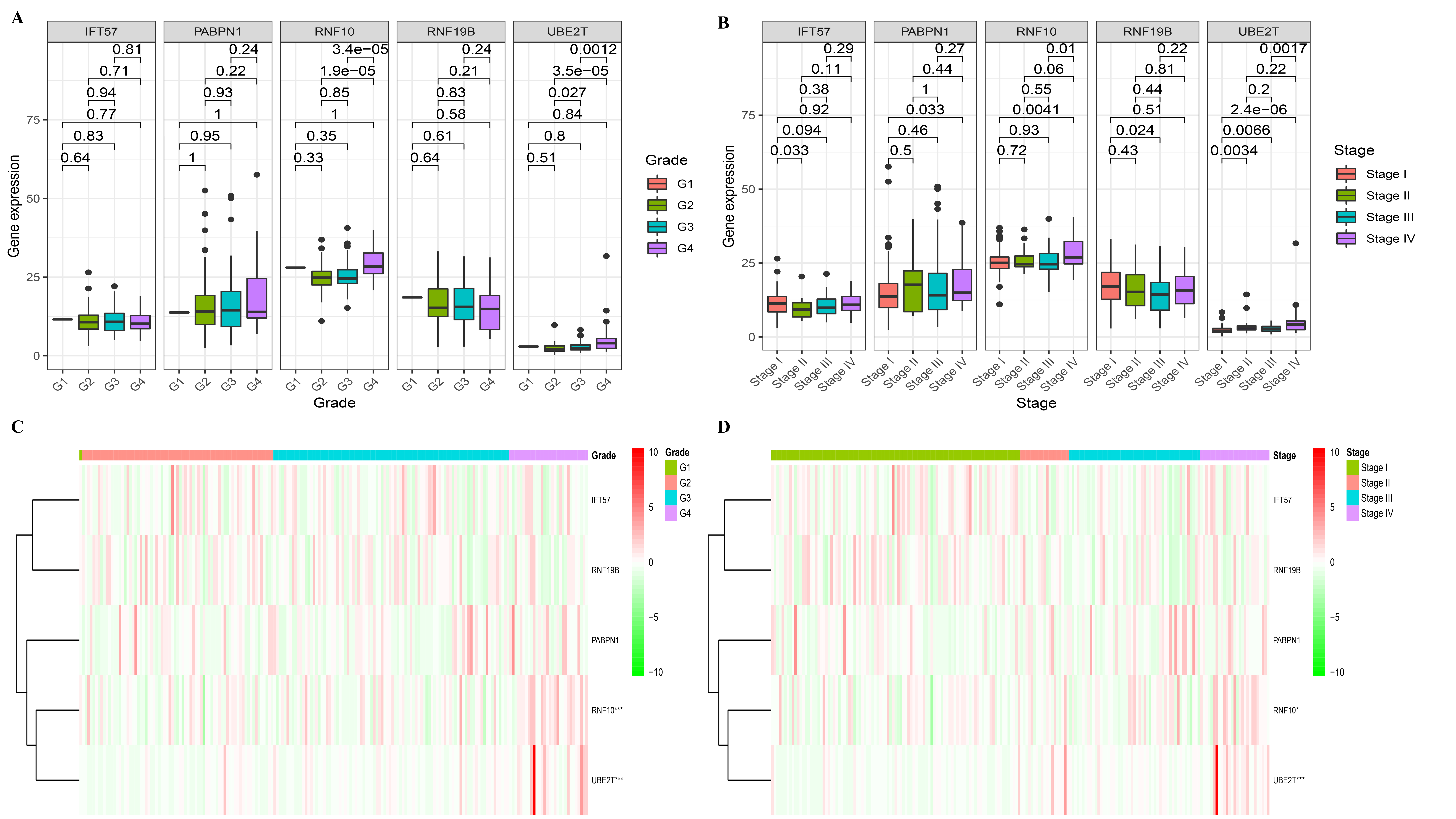
Fig. S6.** The correlation between the hypoxia-related prognostic genes and clinical pathological, such as Grade (A,C) and Stage (B,D), in TCIA-KIRC.

**V. Construction,performance and validation of the combined nomogram**


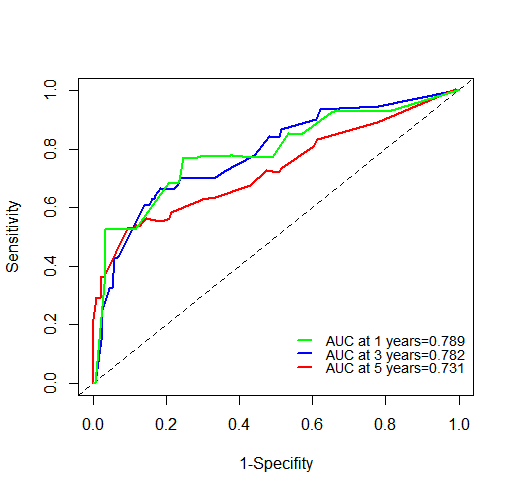


**Fig. S7.** Performance of the combined nomogram. ROC analysis in Cohort 2 to validate the performance of the combined nomogram for 1-,3-and 5-years.


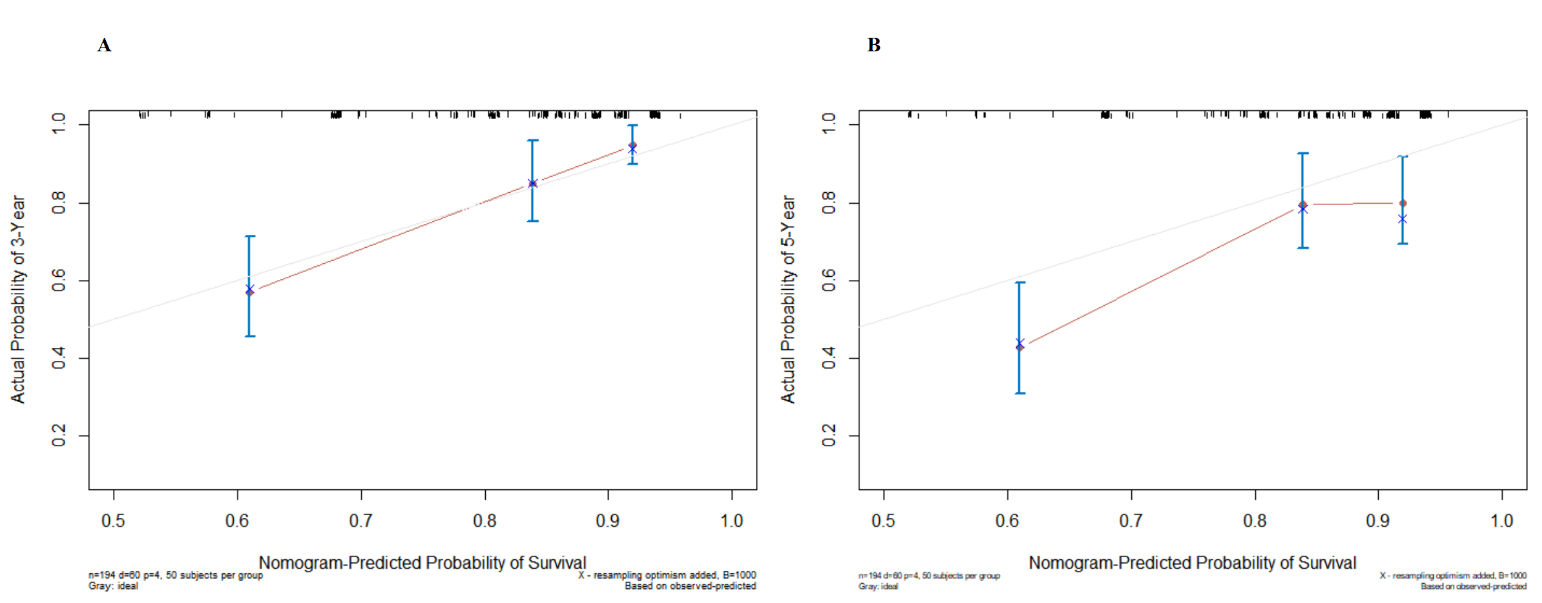


**Fig. S8.** Calibration curves of the combined nomogram for 3-year(A) and 5-year(B) survival in Cohort 2.

**VI.** **R packages we used in this study**

The “glmnet” package was used for LASSO logistic regression. The “rms” package was used for analysis for uni-and multi-variate Cox analysis. The “rms” package was used in the logistic regression analysis and VIF calculation. Calibration curves were established using bootstrapping validation with 1,000 resamples. ROC curves were performed with the “survivalROC” package.
